# Supplementary material for: Targeting prominin2 transcription to overcome ferroptosis resistance in cancer
Source: EMBO Mol Med. 2021 Jul 5;13(8):e13792. doi: 10.15252/emmm.202013792 (PMC8350900; doi:10.15252/emmm.202013792)
Supplement: Supplementary file 1 — Appendix [file EMMM-13-e13792-s003.pdf]

APPENDIX

Targeting prominin2 transcription to overcome ferroptosis resistance in cancer

Caitlin W. Brown, Peter Chhoy, Dimpi Mukhopadhyay, Emmet R. Karner and Arthur M. Mercurio[1]

Department of Molecular, Cell and Cancer Biology, University of Massachusetts Medical School, Worcester, MA

[1]To whom correspondence should be addressed: Arthur M. Mercurio, Department of Molecular, Cell and Cancer Biology, University of Massachusetts Medical School Worcester, MA 01605; e-mail: arthur.mercurio@umassmed.edu; Tel. (508) 856-8676; Fax (508) 856-1310

ORCID ID: 0000-0003-2762-7519

Appendix Table S1: List of statistical method used and p-values.

Figure Number	P-Value	Method
**Figure 1**		
Figure 1A		
MCF10A: RSL3 45 minutes	0.0069	Unpaired student's *t*-test
Figure 1B		
MCF10A: DMSO vs. RSL3	0.00255	Unpaired student's *t*-test
MCF10A: RSL3 vs. RSL3+Fer-1	0.00595	Unpaired student's *t*-test
Figure 1C		
MCF10A: 4HNE 45 minutes	0.0077	Unpaired student's *t*-test
Figure 1D		
MCF10A: EtOH vs. 4HNE	0.0022	Unpaired student's *t*-test
**Figure 2**		
Figure 2C		
MCF10A: DMSO vs. 4HNE	0.0022	Unpaired student's *t*-test
MCF10A: 4HNE vs. 4HNE+BIRB	0.0022	Unpaired student's *t*-test
Figure 2E		
MCF10A: RSL3+DMSO vs. RSL3+BIRB	<0.0001	Unpaired student's *t*-test
**Figure 3**		
Figure 3B		
MCF10A: RSL3 15 minutes	0.0034	Unpaired student's *t*-test
MCF10A: 4HNE 15 minutes	0.0015	Unpaired student's *t*-test
Figure 3C		
MCF10A: Control vs. 4HNE	0.0028	Unpaired student's *t*-test
MCF10A: 4HNE vs. 4HNE+BIRB	0.01	Unpaired student's *t*-test
Figure 3G		
MCF10A: 1 uM RSL3 siControl vs. 1 uM RSL3 siHSF1	<0.0001	Unpaired student's *t*-test
**Figure 4**		
Figure 4A		
MCF10A: DMSO vs. 4HNE	<0.0001	Unpaired student's *t*-test
MCF10A: 4HNE vs. 4HNE+KRIBB11	<0.0001	Unpaired student's *t*-test
MCF10A: RSL3 vs. RSL3+KRIBB11	<0.0001	Unpaired student's *t*-test
Figure 4B		
MCF10A: DMSO vs. 4HNE	0.0093	Unpaired student's *t*-test
Figure 4E		
MCF10A: 0.5 uM RSL3 Control vs. 0.5 uM RSL3 KRIBB11	<0.0001	Unpaired student's *t*-test
MCF10A: 0.5 uM FIN56 Control vs. 0.5 uM FIN56 KRIBB11	<0.0001	Unpaired student's *t*-test
MCF10A: 0.5 uM IKE Control vs. 0.5 uM IKE KRIBB11	<0.0001	Unpaired student's *t*-test

**Figure 5**		
Figure 5A		
Hs578t: 0.5 uM RSL3+DMSO vs. 0.5 uM RSL3+KRIBB11	0.001	Unpaired student's *t*-test
Hs578t: 0.5 uM IKE+DMSO vs. 0.5 uM IKE+KRIBB11	0.003	Unpaired student's *t*-test
Figure 5B		
SF295: 0.25 uM RSL3+DMSO vs. 0.25 uM RSL3+KRIBB11	<0.0001	Unpaired student's *t*-test
SF295: 0.5 uM IKE+DMSO vs. 0.5 uM IKE+KRIBB11	<0.0001	Unpaired student's *t*-test
Figure 5C		
NCI H1975: 0.5 uM RSL3+DMSO vs. 0.5 uM RSL3+KRIBB11	<0.0001	Unpaired student's *t*-test
NCI H1975: 0.5 uM IKE+DMSO vs. 0.5 uM IKE+KRIBB11	<0.0001	Unpaired student's *t*-test
Figure 5E		
MDA-MB-231: 0.25 uM IKE+DMSO vs. 0.25 uM IKE+KRIBB11	<0.0001	Unpaired student's *t*-test
**Figure 6**		
Figure 6B		
Hs578t: Day 7 Vehicle vs. Day 7 IKE+KRIBB11	<0.0001	Two-way ANOVA
**Figure EV1**		
Figure EV1B		
MCF10A: DMSO vs. RSL3	0.0017	Unpaired student's *t*-test
MCF10A: DMSO vs. 4HNE	0.0033	Unpaired student's *t*-test
**Figure EV2**		
Figure EV2B		
Hs578t: 1uM RSL3+DMSO vs. 1uM RSL3+BIRB	<0.0001	Unpaired student's *t*-test
Figure EV2D		
MCF10A: DMSO+RSL3 vs. SB202190+RSL3	<0.0001	Unpaired student's *t*-test
**Figure EV3**		
Figure EV3D		
0.5 uM RSL3: MCF10A+Vector RSL3 vs. MCF10A+Vector RSL3+KRIBB11	<0.0001	Unpaired student's *t*-test
1 uM RSL3: MCF10A+Vector RSL3 vs. MCF10A+Vector RSL3+KRIBB11	<0.0001	Unpaired student's *t*-test
1 uM RSL3: MCF10A+Prominin2 RSL3 vs. MCF10A+Prominin2 RSL3+KRIBB11	0.0078	Unpaired student's *t*-test

2.5 uM RSL3: MCF10A+Vector RSL3 vs. MCF10A+Vector RSL3+KRIBB11	<0.0001	Unpaired student's *t*-test
2.5 uM RSL3: MCF10A+Prominin2 RSL3 vs. MCF10A+Prominin2 RSL3+KRIBB11	0.0064	Unpaired student's *t*-test
5 uM RSL3: MCF10A+Vector RSL3 vs. MCF10A+Vector RSL3+KRIBB11	<0.0001	Unpaired student's *t*-test
Figure EV3E		
MCF10A: siHSF1 RSL3 vs. siHSF1 RSL3+Fer-1	<0.0001	Unpaired student's *t*-test
**Figure EV4**		
Figure EV4B		
MCF10A: IKE vs. IKE+KRIBB11	<0.0001	Unpaired student's *t*-test
MCF10A: IKE vs. IKE+KRIBB11+Fer-1	0.0042	Unpaired student's *t*-test
MCF10A: IKE vs. IKE+KRIBB11+ZVAD-fmk	<0.0001	Unpaired student's *t*-test
Figure EV4C		
MCF10A: 2 uM RSL3 vs. 2 uM RSL3+BIRB	<0.0001	Unpaired student's *t*-test
**Figure EV5**		
Figure EV5B		
MDA-MB-231: 0.25 uM RSL3+DMSO vs. 0.25 uM RSL3+KRIBB11	<0.0001	Unpaired student's *t*-test
