# Supplementary material for: Targeting prominin2 transcription to overcome ferroptosis resistance in cancer
Source: EMBO Mol Med. 2021 Jul 5;13(8):e13792. doi: 10.15252/emmm.202013792 (PMC8350900; doi:10.15252/emmm.202013792)
Supplement: Supplementary file 2 — Expanded View Figures PDF [file EMMM-13-e13792-s001.pdf]

Expanded View Figures

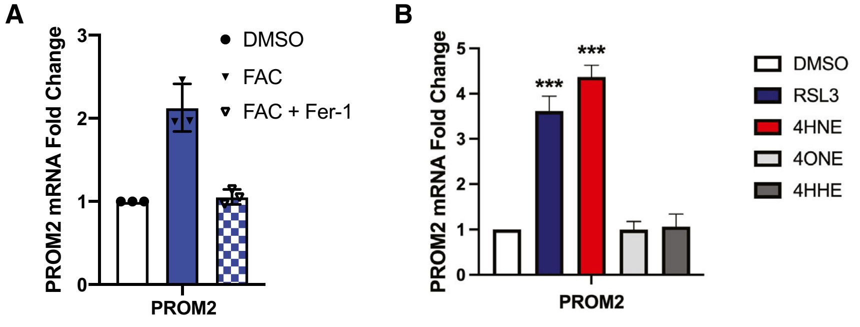

Figure EV1. Assessment of PROM2 mRNA regulation by iron and lipid metabolites.

- A MCF10A cells were pretreated for 15 min with either DMSO or 2 μM fer-1, followed by 60 min with either DMSO, 250 μM FAC, or FAC and fer-1. mRNA was isolated and *PROM2* expression was quantified by qPCR. Shown are three independent experiments with standard deviation (*n* = 3 experiments per group).
- B MCF10A cells were treated for 60 min with either DMSO, 5 μM RSL3, 25 μM 4HNE, 25 μM 4ONE, or 25 μM 4HHE. mRNA was isolated and *PROM2* expression was quantified by qPCR. Shown are three independent experiments with standard deviation (*n* = 3 experiments per group). *P*-values were obtained by unpaired Student's *t*-test with *$P < 0.05$, **$P < 0.01$, ***$P < 0.005$. Exact *P*-values are reported in Appendix Table S1.

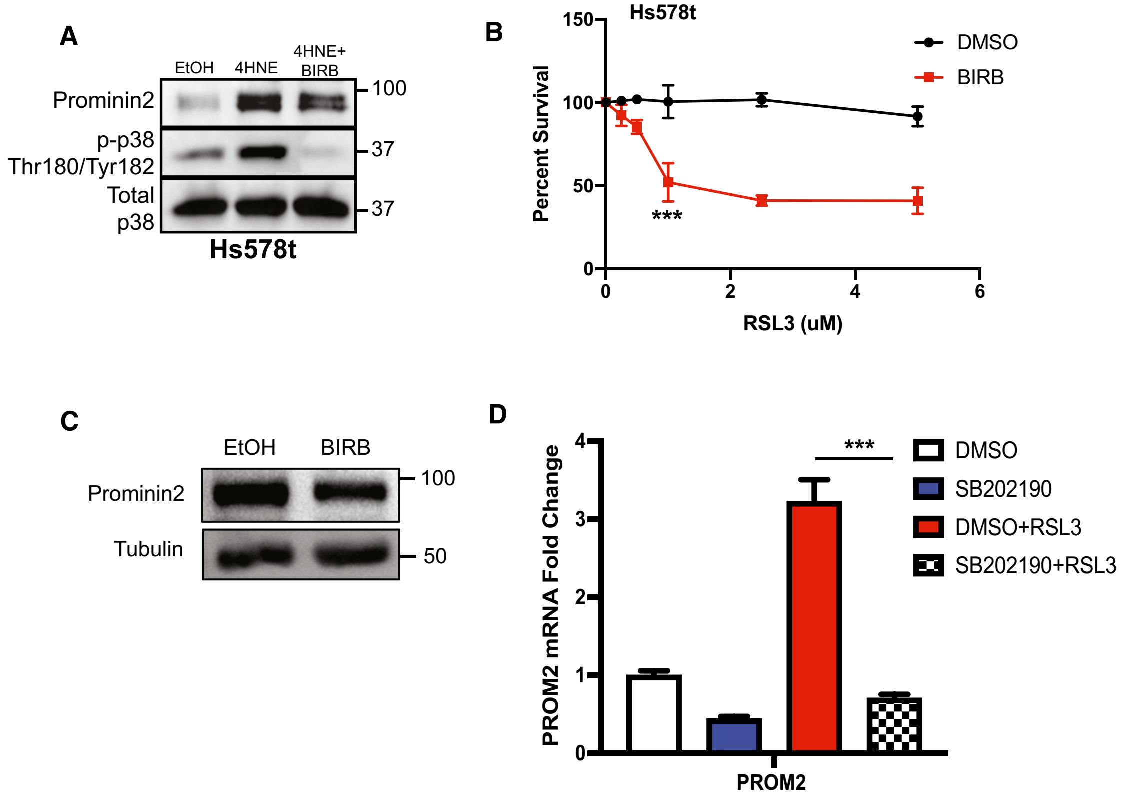

Figure EV2.

Figure EV2. Regulation of prominin2 by p38 MAPK.

A Hs578 t cells were pretreated for 15 min with either EtOH or 10 μM BIRB, followed by 60 min with either EtOH, 25 μM 4HNE, 5 μM RSL3, 4HNE, and BIRB or RSL3 and BIRB. Isolated protein was assessed by immunoblotting for prominin2, phospho-p38 (Thr180/Tyr182), and total p38. Shown is one replicate of three independent experiments (n = 3).

B Hs578t cells were treated with RSL3 with either DMSO or 10 μM BIRB. Cells were assessed for viability after 24 h. Absorbance was normalized to DMSO control. Shown are three independent replicates with standard deviation (n = 3 experiments per group). P-values were obtained by unpaired Student's t-test with $^*P < 0.05$, $^{**}P < 0.01$, $^{***}P < 0.005$. Exact P-values are reported in Appendix Table S1.

C MCF10A cells were pretreated for 15 min with either EtOH or 10 μM BIRB, followed by 60 min with either EtOH or 10 μM BIRB. Isolated protein was assessed by immunoblotting for prominin2 and β-tubulin expression. Shown is one replicate of three independent experiments (n = 3).

D MCF10A cells were pretreated for 15 min with either DMSO or 10 μM SB202190, followed by 60 min with either DMSO, 5 μM RSL3, 10 μM SB202190, or RSL3 and SB202190. mRNA was isolated and *PROM2* expression was quantified by qPCR. Shown are three independent experiments with standard deviation (n = 3 experiments per group). P-values were obtained by unpaired Student's t-test with $^*P < 0.05$, $^{**}P < 0.01$, $^{***}P < 0.005$. Exact P-values are reported in Appendix Table S1.

Figure EV3. Increasing prominin2 expression protects against HSF1 inhibition.

A Hs578 t cells were pretreated for 15 min with either EtOH or 10 μM BIRB, followed by 60 min with either EtOH, 25 μM 4HNE, 5 μM RSL3, 4HNE, and BIRB or RSL3 and BIRB. Isolated protein was assessed by immunoblotting for prominin2, phospho-HSF1 (S326), total HSF1, phospho-p38 (Thr180/Tyr182), and total p38 expression. Shown is one replicate of three independent experiments (n = 3).

B Hs578t cells were plated on slides pre-coated with laminin. Cells were treated for 60 min in either EtOH or 25 μM 4HNE. Cells were stained for total HSF1 and counterstained with DAPI. Images were taken at 20× magnification. Scale bar is 50 μm. Shown is one replicate of three independent experiments (n = 3).

C Cell lysates from MCF10A cells transfected with either the prominin2 expression construct or the vector control were assessed by immunoblotting for prominin2 and b-actin expression. Shown is one replicate of three independent experiments (n = 3).

D MCF10A cells were transfected with either a prominin2 expression construct or a vector control prior to treatment with either DMSO, 10 μM KRIBB11, RSL3, or KRIBB11 and RSL3 at the range of concentrations shown for 24 h, and the number of viable cells was quantified. Shown is representative experiment of three independent replicates (n = 3 experiments per group). Data are plotted as the mean + SD. P-values were obtained by unpaired Student's t-test with $^*P < 0.05$, $^{**}P < 0.01$, $^{***}P < 0.005$. Exact P-values are reported in Appendix Table S1.

E siControl or siHSF1-treated MCF10A were maintained for 24 h in the presence of either DMSO, 2 μM ferrostatin-1, 5 μM RSL3, or ferrostatin-1, and RSL3, and the number of viable cells was quantified. Shown is representative experiment of three independent replicates (n = 3 experiments per group). Data are plotted as the mean + SD. P-values were obtained by unpaired Student's t-test with $^*P < 0.05$, $^{**}P < 0.01$, $^{***}P < 0.005$. Exact P-values are reported in Appendix Table S1.

 © 2021 The Authors

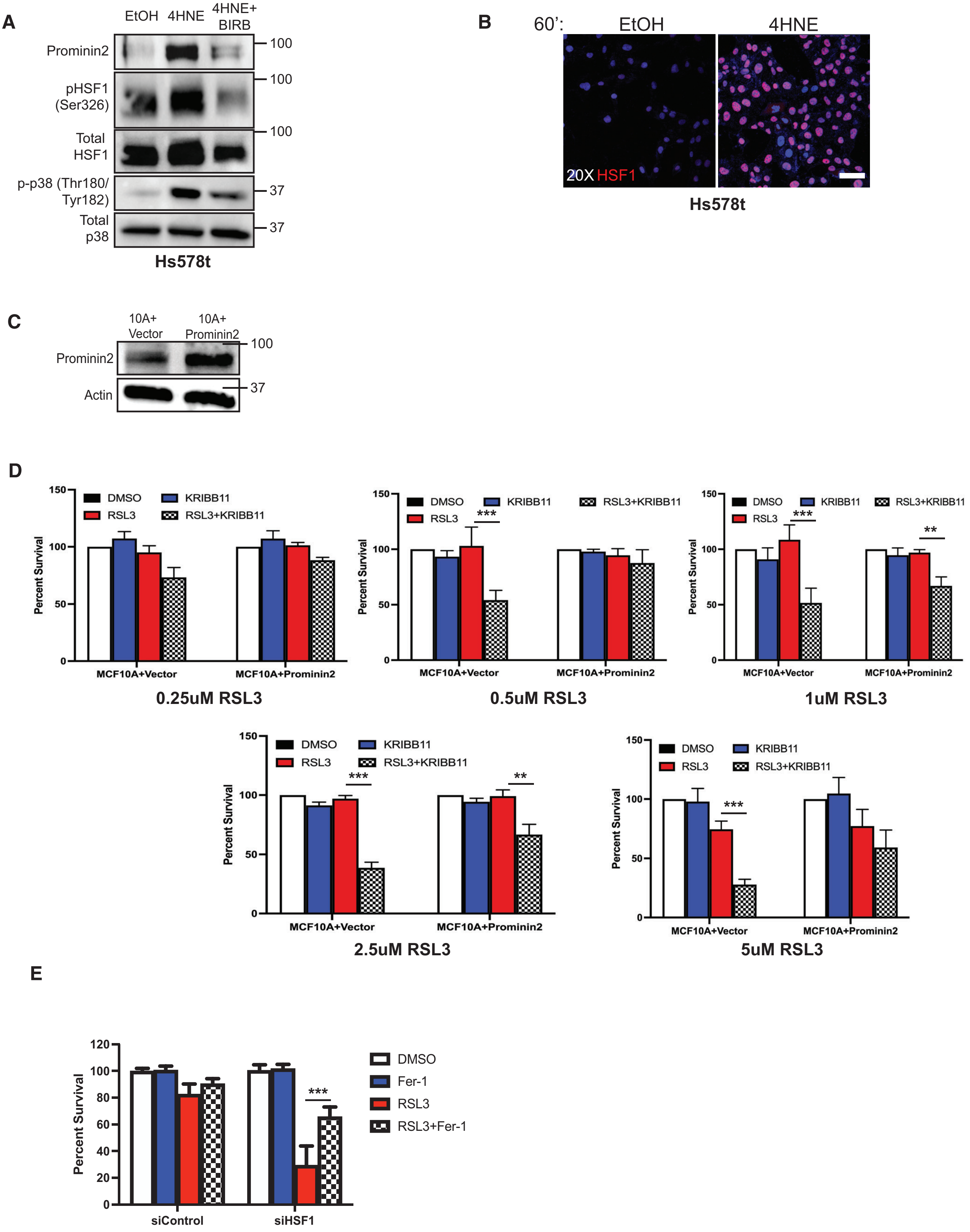
A
EtOH
4HNE
4HNE+ BIRB
Prominin2
pHSF1 (Ser326)
Total HSF1
p-p38 (Thr180/ Tyr182)
Total p38
100
100
100
37
37
Hs578t
B
60':
EtOH
4HNE
20X HSF1
Hs578t
C
10A+ Vector
10A+ Prominin2
Prominin2
Actin
100
37
D
DMSO
KRIBB11
RSL3
RSL3+KRIBB11
Percent Survival
MCF10A+Vector
MCF10A+Prominin2
0.25uM RSL3
0.5uM RSL3
1uM RSL3
2.5uM RSL3
5uM RSL3
E
Percent Survival
siControl
siHSF1
DMSO
Fer-1
RSL3
RSL3+Fer-1

Figure EV3.

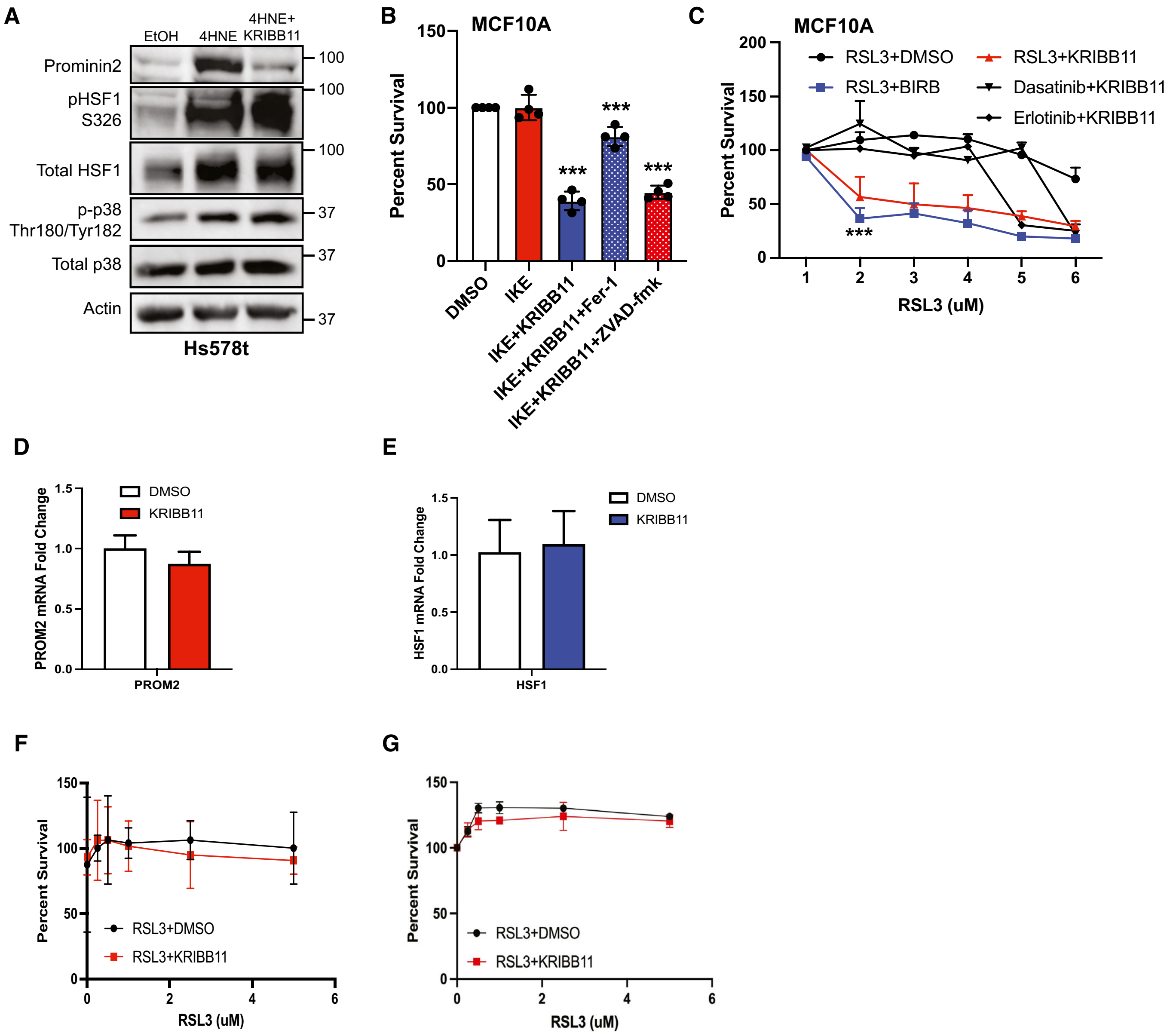

Figure EV4. Additional data on pathway inhibition.

A Hs578t cells were treated for 60 min with either EtOH, 25 µM 4HNE, 5 µM RSL3, 4HNE, and 10 µM KRIBB11 or RSL3 and KRIBB11. Isolated protein was assessed by immunoblotting for prominin2, phospho-HSF1 (Ser326), total HSF1, phospho-p38 (Thr180/Tyr182), total p38, and β-actin expression. Shown is one replicate of three independent experiments ($n = 3$).

B MCF10A cells were treated with either DMSO, 1 µM IKE, IKE + 10 µM KRIBB11, IKE + KRIBB11 + 2 µM Fer-1, or IKE + KRIBB11 + 25 µM ZVAD-fmk. Cells were assessed for viability after 24 h. Absorbance was normalized to DMSO control. Shown are four independent replicates with standard deviation ($n = 3$ experiments per group). *P*-values were obtained by unpaired Student's *t*-test with $^{*}P < 0.05$, $^{**}P < 0.01$, $^{***}P < 0.005$. Exact *P*-values are reported in Appendix Table S1.

C MCF10A cells were treated with RSL3 at the range of concentrations shown with either DMSO, 10 µM BIRB, 10 µM KRIBB11, 100 nM dasatinib, or 25 µM erlotinib. Cells were assessed for viability after 24 h. Absorbance was normalized to DMSO control. Shown are three independent replicates with standard deviation ($n = 3$ experiments per group). *P*-values were obtained by unpaired Student's *t*-test with $^{*}P < 0.05$, $^{**}P < 0.01$, $^{***}P < 0.005$. Exact *P*-values are reported in Appendix Table S1.

D MCF10A cells were treated for 60 min with either DMSO or 10 µM KRIBB11. mRNA was isolated, and *PROM2* expression was quantified by qPCR. Shown are three independent experiments with standard deviation ($n = 3$ experiments per group).

E MCF10A cells were treated for 60 min with either DMSO or 10 µM KRIBB11. mRNA was isolated, and *HSF1* expression was quantified by qPCR. Shown are three independent experiments with standard deviation ($n = 3$ experiments per group).

F HMLE cells were treated with RSL3 at the range of concentrations shown in combination with either DMSO or 10 µM KRIBB11. Cells were assessed for viability after 24 h (using crystal violet). Absorbance was normalized to DMSO control. Shown are three independent replicates with standard deviation ($n = 3$ experiments per group).

G S1 cells were treated with RSL3 at the range of concentrations shown in combination with either DMSO or 10 µM KRIBB11. Cells were assessed for viability after 24 h (using crystal violet). Absorbance was normalized to DMSO control. Shown are three independent replicates with standard deviation ($n = 3$ experiments per group).

© 2021 The Authors

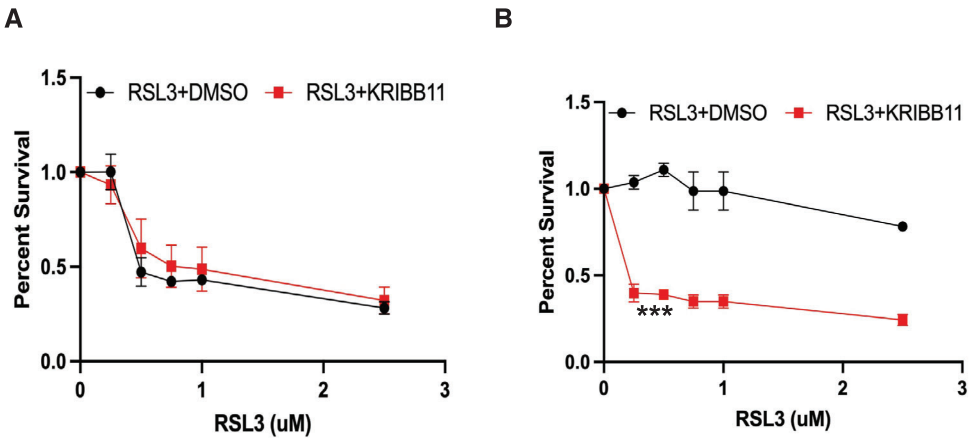

Figure EV5. Acquired resistance to RSL3 can be overcome by HSF1 inhibition.

A MDA-MB-231 RSL3-sensitive cells were treated with RSL3 at the range of concentrations shown in combination with either DMSO or 10 μM KRIBB11. Cells were assessed for viability after 24 h. Absorbance was normalized to DMSO control. Shown are three independent replicates with standard deviation (n = 3 experiments per group).

B MDA-MB-231 RSL3-resistant cells were treated with RSL3 at the range of concentrations shown in combination with either DMSO or 10 μM KRIBB11. Cells were assessed for viability after 24 h. Absorbance was normalized to DMSO control. Shown are three independent replicates with standard deviation (n = 3 experiments per group). P-values were obtained by unpaired Student's t-test with *$P < 0.05$, **$P < 0.01$, ***$P < 0.005$. Exact P-values are reported in Appendix Table S1.
